# Effects of psychological skills training on self-control, perceived physical self-defense capacity, and mental toughness in elite female wrestlers: a randomized controlled trial

**DOI:** 10.3389/fpsyg.2026.1956445

**Published:** 2026-09-30

**Authors:** Varol Tutal, Okan Demir, Oktay Şahin, Ahmet Yılmaz Albayrak, Mehmet Bayansalduz, Recep Nur Uzun, Faik Öz, Yunus Şahinler, Levent Ceylan, Coşkun Yılmaz

**Affiliations:** 1 Faculty of Health Sciences, Malatya Turgut Özal University, Malatya, Türkiye; 2 Şiran Mustafa Beyaz Vocational School, Gümüşhane University, Gümüşhane, Türkiye; 3 Faculty of Sport Sciences, Gümüşhane University, Gümüşhane, Türkiye; 4 Faculty of Sports Sciences, Topkapı University, İstanbul, Türkiye; 5 Yaşar Doğu Faculty of Sport Sciences, Ondokuz Mayıs University, Samsun, Türkiye; 6 Faculty of Sport Sciences, Avrasya University, Trabzon, Türkiye; 7 Faculty of Sport Sciences, İstanbul Gelisim University, İstanbul, Türkiye; 8 Faculty of Sport Sciences, Hitit University, Çorum, Türkiye

**Keywords:** athlete development, evidence-based interventions, performance optimization, psychological skills training, sport psychology, wrestling

## Abstract

**Background:**

Self-control, self-defense self-efficacy, and mental resilience are fundamental psychological determinants of performance and competitive endurance in combat sports. However, scientific evidence on structured, evidence-based psychological intervention programs for professional female wrestlers remains limited. This study examined the effects of a psychological skills training (PST)-based intervention on self-control, self-defense self-efficacy, and mental resilience in professional female wrestlers.

**Methods:**

A randomized controlled pre-test–post-test design was employed. Thirty-six professional female wrestlers (aged 18–25) were randomly assigned to a PST group (*n* = 18) or a control group (CG; *n* = 18). The PST group completed an 8-week structured psychological intervention alongside regular training, while the CG continued routine training only. Self-control, self-defense self-efficacy, and mental resilience were assessed pre- and post-intervention using valid and reliable psychometric scales. Data were analyzed via repeated-measures ANOVA, with effect sizes calculated.

**Results:**

Significant group × time interactions were observed across multiple outcomes. A highly significant interaction emerged for perceived physical self-defense capacity (SP; *F*(1,34) = 56.525, *p* < 0.001, ηp^2^ = 0.624) and for DP scores (*F* (1,34) = 9.360, *p* = 0.004, ηp^2^ = 0.216). Among self-control subscales, significant interactions were found for inhibition (*F* (1,34) = 8.172, *p* = 0.007, ηp^2^ = 0.194) and, more strongly, for initiation (*F* (1,34) = 14.157, *p* < 0.001, ηp^2^ = 0.294). For mental resilience, highly significant interactions were detected for confidence (*F* (1,34) = 59.976, *p* < 0.001, ηp^2^ = 0.638), control (*F* (1,34) = 32.486, *p* < 0.001, ηp^2^ = 0.489), and persistence (*F* (1,34) = 32.709, *p* < 0.001, ηp^2^ = 0.490). Overall, the intervention demonstrated medium-to-high effect sizes across psychological performance indicators.

**Conclusion:**

These findings suggest that PST may serve as a useful complementary approach for enhancing self-control, perceived physical self-defense capacity, and mental toughness in professional female wrestlers. However, results should not be interpreted as evidence of improved competitive wrestling performance, technical success, or objectively measured physical self-defense ability, as these outcomes were not directly assessed in this study.

## 1 Introduction

Combat sports are increasingly recognized not only as performance-oriented activities but also as multidimensional contexts with implications for physical health, psychological resilience, self-regulation, self-esteem, and social development. However, these potential benefits coexist with sport-specific demands and risks, including injury, psychological stress, anxiety, burnout, and negative self-perceptions. Accordingly, contemporary approaches emphasize evidence-based, safe, ethical, and context-sensitive practices that can support both performance and wellbeing across combat-sport settings (Ciaccioni et al., 2023, 2025a,b,^2026^; Okudan et al., 2025; Özkan et al., 2026). Recent work has further highlighted the importance of psychological preparation, self-regulatory resources, instructor practices, and structured training environments in determining whether participation in combat sports produces sustainable health and developmental benefits (Ciaccioni et al., 2025a,b, 2026).

Wrestling provides a particularly demanding context because of intense physical contact, high training loads, weight-management demands, and competitive pressure (Okudan et al., 2025; Özkan et al., 2026). Female wrestlers may additionally encounter challenges related to performance expectations, weight control, injury risk, and social pressures (Durukan et al., 2025; Nicholls et al., 2009; Seker et al., 2024). At the same time, participation in combat sports may provide opportunities to develop confidence, perceived competence, autonomy, and psychological resources relevant to agency and empowerment (Ciaccioni et al., 2025a; Yıkılmaz et al., 2025). Within this context, self-control–the capacity to regulate impulses, behavior, and goal-directed actions–is an important self-regulatory resource (Baumeister et al., 2007). Self-control is particularly relevant to combat-sport participation because athletes must continuously regulate attention, emotions, behavior, and responses to demanding situations (Brown and Fletcher, 2017; Park and Jeon, 2023; Şahin et al., 2025; Vealey, 2023; Wang et al., 2025). Evidence from combat-sport and martial-arts research also suggests that structured training can be associated with self-regulation, psychological resilience, and broader psychological functioning (Ciaccioni et al., 2023; Harwood-Gross et al., 2021; Köroğlu et al., 2025; Ng-Knight et al., 2021; Şahin et al., 2025).

Perceived physical self-defense capacity represents another relevant psychological construct. In the present study, this construct refers to athletes’ perceived ability to respond effectively to threatening physical situations and is therefore conceptually related to self-efficacy and perceived competence rather than objectively demonstrated self-defense skill (Bandura, 1997; Yıkılmaz et al., 2025). This distinction is important because perceived capability may influence confidence, adaptive responses, and psychological functioning, while actual self-defense performance requires direct behavioral assessment. Mental toughness represents a further psychological resource, generally characterized by confidence, persistence, and psychological control under demanding or stressful conditions (Clough et al., 2002; Gucciardi et al., 2017). Importantly, mental toughness should not be equated with mental health; rather, it may represent a psychological resource that supports goal-directed functioning while interacting with broader wellbeing processes (Gucciardi et al., 2017; Vealey, 2023; Wang et al., 2025).

Psychological skills training (PST) provides a structured approach for developing such resources through techniques including goal setting, imagery, self-talk, relaxation, attentional control, and related self-regulatory strategies (Brown and Fletcher, 2017; Park and Jeon, 2023; Vealey, 2023; Weinberg and Gould, 2024). These techniques may influence psychological functioning through complementary pathways, including behavioral monitoring and goal regulation, mental rehearsal and perceived competence, arousal regulation, attentional control, and task-focused coping (Vealey, 2023; Weinberg and Gould, 2024). Evidence from meta-analyses, systematic reviews, and recent sport-psychology research supports the potential value of psychological and psychosocial interventions for athletic performance and athlete mental well-being (Andreato et al., 2022; Brown and Fletcher, 2017; Lange-Smith et al., 2023; Reinebo et al., 2023; Vealey, 2023; Wang et al., 2025). Within combat sports, psychological preparation and mental training have likewise been identified as potentially relevant to self-regulation, resilience, self-efficacy, and psychological functioning (Ciaccioni et al., 2023; Harwood-Gross et al., 2021; Köroğlu et al., 2025; Moore et al., 2023; Ng-Knight et al., 2021; Oguntuase and Sun, 2022; Yıkılmaz et al., 2025; Zheng et al., 2026).

Nevertheless, the effectiveness and sustainability of psychological interventions cannot be considered independently of their implementation context. Recent conceptual and editorial work on combat-sport wellbeing emphasizes that positive psychological outcomes depend on structured training environments, appropriate instructor practices, safety norms, relational and ethical considerations, access to self-regulatory resources, and context-sensitive implementation (Ciaccioni et al., 2025a,b, 2026). Therefore, PST should be considered a complementary component of athlete development rather than a substitute for technical, physical, or coaching practices. This perspective is particularly relevant to female combat-sport athletes, for whom psychological preparation may have implications extending beyond competition to perceived competence, agency, resilience, and psychological well-being (Ciaccioni et al., 2025a,b; Yıkılmaz et al., 2025).

Despite growing evidence supporting psychological interventions in sport and combat-sport contexts, relatively few randomized controlled studies have simultaneously examined self-control, perceived physical self-defense capacity, and mental toughness following a structured PST intervention in professional female wrestlers (Andreato et al., 2022; Brown and Fletcher, 2017; Lange-Smith et al., 2023; Reinebo et al., 2023). Moreover, the extent to which such an intervention influences psychological resources relevant to wellbeing within a structured wrestling environment remains insufficiently understood. Therefore, the present study aimed to investigate the effects of an 8-week structured PST program on self-control, perceived physical self-defense capacity, and mental toughness in professional female wrestlers. In accordance with the multidimensional structure of the SMTQ-14, mental toughness was considered an overall construct comprising confidence, persistence, and control. We hypothesized that professional female wrestlers participating in the PST program would demonstrate greater improvements in self-control, perceived physical self-defense capacity, and overall mental toughness than those receiving routine training alone. Because psychological wellbeing, objective self-defense performance, and competitive wrestling performance were not directly assessed, conclusions regarding these broader outcomes were considered beyond the direct scope of the present study.

## 2 Materials and methods

### 2.1 Participants

This study was designed as a parallel, two-arm, pre-test-post-test randomized controlled trial conducted in accordance with the CONSORT 2025 guidelines (Hopewell et al., 2025). Prior to participation, all volunteers were informed in detail about the study procedures, and written consent was obtained in accordance with the ethical principles of the Declaration of Helsinki. All participants were informed both verbally and in writing prior to enrollment that they could withdraw from the study at any time without giving a reason and without any consequences for their routine medical care. Ethical approval was granted by the Scientific Research Ethics Committee of Hitit University (Date: 29 August 2025; No: 2025/0472).

This study was designed to examine the effectiveness of a structured psychological skills training-based psychological performance intervention program aimed at developing mental resilience, self-control, and self-defence self-efficacy in female wrestlers aged 18–25. Thirty-six healthy female wrestlers were included in the study. An *a priori* power analysis was executed via G*Power (Version 3.1.9.7) to estimate the minimum sample size required to detect robust statistical differences. Based on self-control (Ciaccioni et al., 2025a) as the primary outcome measure, based on an anticipated large effect size (partial eta squared, ηp^2^) = 0.14, corresponding to Cohen’s *f* = 0.40, a conventional Type I error rate of α = 0.05, and a desired power (1-β) of 0.95, the algorithm determined that 15 participants per cohort were necessary to avoid Type II errors. Accounting for an expected 20% attrition rate typical in longitudinal sports science interventions, 18 participants were recruited per group, yielding a final sample of *n* = 36. The study was designed as a randomized controlled trial. Participants were randomly assigned in a 1:1 ratio to the psychological skills training (PST) group (*n* = 18) or the control group (CG; *n* = 18). All 36 randomized participants completed the intervention and post-intervention assessments, and no participants were lost to follow-up. Therefore, the final analysis included all 36 randomized participants. Randomization was performed using a computer-generated random sequence by an independent researcher not involved in the assessments or intervention program. Assignment concealment was ensured using sequentially numbered, sealed, opaque envelopes opened after baseline measurements.

The PST group participated in a psychological skills training program in addition to their wrestling training program, while the control group continued with their regular training routines. Randomization was performed using a computer-generated random sequence created by an independent researcher not involved in the assessments or intervention program. Assignment concealment was ensured using sequentially numbered, sealed, opaque envelopes opened after baseline measurements. However, to minimize detection and analysis bias, outcome assessors and the statistician responsible for data analysis were blinded to group assignment.

Initially, forty female wrestlers were assessed for eligibility. Four participants were excluded from the study: two declined to participate, one was excluded because of personal reasons or program conflicts, and one was excluded for health-related reasons. The remaining thirty-six participants were randomly assigned to two groups: the PST group and the CG group, each comprising eighteen female wrestlers. To determine which group subjects in the sample would be assigned to, numbers from 1 to 36 were randomly assigned to the two groups using a computer program.^1^ All stages of the study were conducted under the supervision of a physician to ensure participant safety.

### 2.2 Experimental design

Healthy women participating in the study were asked to visit the laboratory three times. During the first visit, they were provided with information about the training procedures and scales. Each participant received a detailed explanation of the psychological skills training procedure. During the second visit, which took place 1 week later, pre-intervention measurements were taken and the values recorded. At the end of the 8-week training program, final measurements were taken during the third and final visits (Figure 1).

**FIGURE 1:**
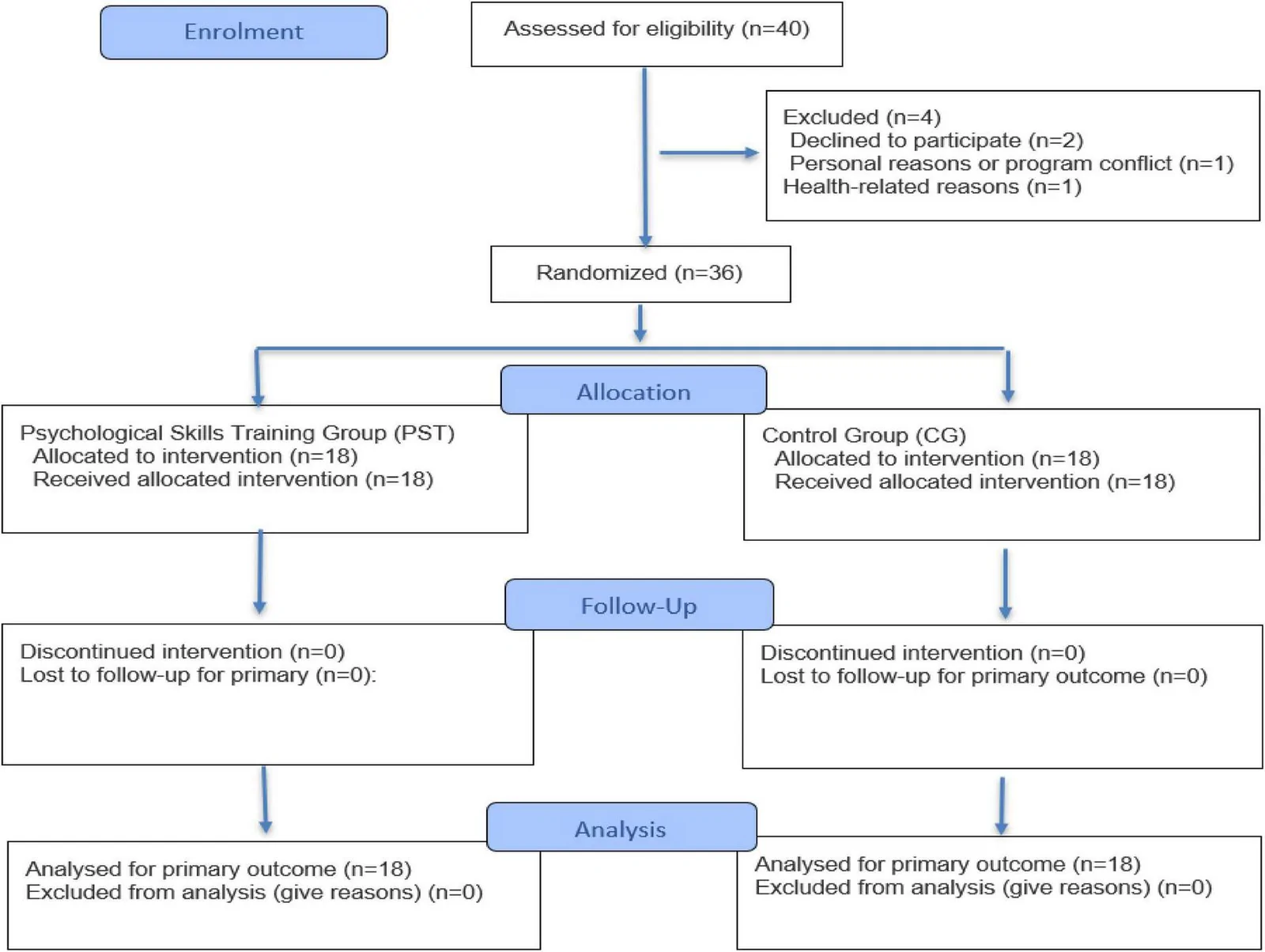
Experimental design.

### 2.3 Body composition measurement

In the study, body composition was measured using the Gaia 359 Plus BodyPass bioelectrical impedance analyzer to demonstrate that the participants had similar body characteristics. The participants’ heights and body weights were determined using the Gaia 359 Plus BodyPass (Table 1).

**TABLE 1:** Descriptive.

Parameters↓	PST (n:18)		CG (n:18)	
	Mean	S. D	Mean	S. D
Age	19.47	1.23	19.57	1.44
Height	162.39	5.18	163.22	5.14
Weight	58.33	6.96	59.28	5.78
BMI	22.48	2.23	22.24	1.76

### 2.4 Short multidimensional self-control scale (BMSCS)

Developed by Nilsen et al. (2020) and subsequently adapted into Turkish by Koç et al. (2023), the scale consists of eight items and two subscales (Inhibition and Initiation) (Koç et al., 2023; Nilsen et al., 2020). The Initiation subscale comprises items 2, 4, 5, and 8, while the Inhibition subscale comprises items 1, 3, 6, and 7. To ensure consistency and comparability, items 1, 3, 5, and 7 are reverse-scored. The scale is a five-point Likert-type scale. In this study, the Cronbach’s alpha reliability coefficient was calculated as 0.795 for the Initiation subscale and 0.768 for the Inhibition subscale.

### 2.5 Physical self-defense scale for women

Developed by Uyar (2022), the scale consists of 12 items and two subscales: self-defense against dangerous physical attacks (DP) and self-defense against simple physical attacks (SP; Uyar, 2022). The scale does not contain any items that can be reverse-scored. This five-point Likert-type scale, consisting of two sub-dimensions and 12 items, has a minimum possible score of 12 and a maximum possible score of 60. The first sub-dimension, covering self-defense against dangerous physical attacks, consists of six items. A high score on this dimension indicates that women have a high level of self-defense ability against physical attacks they perceive as more dangerous. The remaining sub-dimension assesses women’s self-defense capacity in the context of physical attacks they perceive as less dangerous and consists of six items. A high score on this sub-dimension indicates that women have a strong self-defense capacity against physical attacks they perceive as less dangerous. In this study, the alpha coefficient for the dangerous attack sub-dimension was 0.812, and for the simple attack sub-dimension, 0.826. The scale was found to have very high reliability results, with 0.80 ≤ α < 1.00.

SP and DP scores were calculated as the sum of the six items belonging to each respective subscale. No item-level averaging, score transformation, imputation, or rescaling was applied. The scale contained no reverse-scored items. All participants had complete item-level data; therefore, no missing-item procedure was required.

### 2.6 Sport Mental Toughness Questionnaire (SMTQ)

The Sport Mental Toughness Questionnaire (SMTQ-14), developed by Sheard et al. (2009) to assess athletes’ levels of mental toughness, consists of a total of 14 items (Sheard, 2009). The questionnaire, which assesses overall mental toughness as well as three sub-dimensions (confidence, persistence, and control), is a four-point Likert scale. The Sport Mental Toughness Questionnaire has a reliability coefficient suitable for use in this sample. When evaluated in terms of subscales, the alpha coefficient (α) for the confidence subscale was found to be 0.786, for persistence, α: 0.774, and for control, α: 0.816.

### 2.7 Psychological skills training

The intervention consisted of an 8-week structured psychological skills training (PST) programme specifically developed for professional female wrestlers based on contemporary evidence-based sport psychology principles and established PST frameworks proposed by Vealey and subsequently expanded by Weinberg and Gould. The programme followed the classical three-stage PST model comprising education, acquisition, and practice, in which athletes first develop an understanding of psychological skills, subsequently acquire these skills through structured exercises, and finally integrate them into sport-specific training and competitive situations to facilitate automaticity and transfer to performance (Park and Jeon, 2023; Vealey, 2023; Weinberg and Gould, 2024). The intervention was designed to improve psychological characteristics that are highly relevant to wrestling performance, including self-control, self-defense self-efficacy, emotional regulation, attentional control, confidence, and mental resilience (Park and Jeon, 2023; Vealey, 2023). Rather than relying on a single psychological technique, the programme integrated multiple evidence-based PST components, including mental profiling, goal setting, arousal regulation, diaphragmatic breathing, progressive muscle relaxation, imagery based on the PETTLEP model, cognitive restructuring, positive self-talk, attentional control strategies, pre-performance routines, body-image regulation during weight management, and coping strategies for competitive stress. This multidimensional approach reflects current recommendations suggesting that integrated PST programmes produce more comprehensive psychological adaptations than isolated mental skill interventions (Brown and Fletcher, 2017; Park and Jeon, 2023; Wang et al., 2025).

The programme consisted of one 30-min supervised theoretical session each week, immediately followed by daily 5–10-min micro-interventions integrated into routine wrestling practice. Integrating psychological exercises directly into regular training sessions was intended to maximize ecological validity and facilitate the transfer of psychological skills to realistic performance environments. Each week targeted a specific psychological competency while progressively building upon skills acquired during previous sessions. The weekly progression moved from baseline psychological profiling and goal setting toward increasingly complex skills including autonomic regulation, imagery, cognitive restructuring, attentional control under fatigue, management of weight-cutting stress, and finally the integration of all psychological skills during simulated competition scenarios. The complete intervention protocol is presented in Table 2.

**TABLE 2:** The 8-week psychological skills training (PST) protocol for professional female wrestlers.

Week / theme	Theoretical session (30 min)	Daily micro-interventions (5–10 min/day) and contextual integration	Targeted psychometric outcome
Week 1: Mental profiling and baseline	Introduction to PST; Collaborative SWOT analysis based on performance profiling.	Mon-Sat: Post-training digital reflection log. Completion of state-anxiety measures.	Baseline assessment and self-awareness
Week 2: Goal setting and motivation	Process-oriented vs. outcome-oriented goals; SMART criteria in combat sports.	Pre-training: Defining 1 technical and 1 tactical daily micro-goal. Post-training: Self-evaluation scale (1–10).	Task orientation and self-efficacy
Week 3: Arousal regulation	Autonomic nervous system control; Diaphragmatic breathing and progressive muscle relaxation (PMR).	During mat session: 4-7-8 breathing technique during 2-min recovery intervals between sparring rounds.	Heart rate variability (HRV) and somatic anxiety
Week 4: Imagery and visualization	Neurocognitive basis of visualization; Utilizing the PETTLEP model for motor skill enhancement.	Tue: Proactive imagery (kinaesthetic execution of elite throws). Thu: Reactive imagery (crisis management under a deficit).	Motor program consolidation
Week 5: Cognitive restructuring	Self-talk taxonomy (instructional vs. motivational); Identifying and reframing cognitive distortions.	Pre-bout Simulation: Replacing negative triggers (“I cannot defend this takedown”) with cue words (“Hips low, counter”).	Cognitive anxiety and attentional focus
Week 6: Attentional focus and routines	Nideffer’s attentional model: Designing pre-performance and closed-loop routines under fatigue.	Pre-sparring: Execution of a strict 3-step physical and psychological routine (shoelace tie, deep breath, cue word).	Perceptual-motor control
Week 7: Rapid weight loss and body image	Managing the psychophysiological stressors of acute dehydration and weight-cutting anxiety.	Daily: Gratitude and self-compassion affirmations during caloric restriction. Cognitive reframing of hunger as readiness.	Mood state regulation and cortisol control
Week 8: Competitive stress integration	Simulation of elite tournament pressure; Coping under adversity and referee bias.	Sat simulation: Shadow wrestling with auditory crowd distraction. Visualizing gold-medal bout scenarios.	Peak resilience and emotional regulation

PST, psychological skills training; PMR, progressive muscle relaxation; PETTLEP, physical, environment, task, timing, learning, emotion, perspective framework.

To ensure intervention fidelity, all sessions were delivered according to a standardized manual by an experienced sport psychologist working in collaboration with the wrestling coaching staff. Coaches documented weekly implementation reports, including session attendance, participant engagement, completion of daily micro-interventions, and any protocol deviations. Attendance was recorded for each session, and intervention adherence was calculated as the proportion of completed sessions relative to the total number of scheduled sessions. All sessions were delivered by the same psychologist, who followed the predefined PST protocol. No deviations from the intervention protocol were recorded during the study period, resulting in a high overall intervention adherence rate of 94.0%. This high level of adherence indicates that the intervention was well tolerated and consistently implemented throughout the study period. Using a single, consistent practitioner also helped maintain consistent intervention delivery across participants and sessions.

Furthermore, participants were informed that the intervention was designed to support training preparation rather than being explicitly focused on improving the study outcomes, thereby reducing potential expectancy effects. Overall, the intervention was designed to reflect the psychological demands of elite wrestling by embedding psychological skill development within routine technical and tactical practice rather than delivering isolated classroom-based instruction. Such an ecologically integrated approach is increasingly recommended in contemporary applied sport psychology, as it facilitates skill acquisition and enhances transfer to competition, thereby potentially maximizing the practical viability of psychological skills training within elite wrestling environments.

### 2.8 Statistical analyses

All statistical analyses were performed using IBM SPSS Statistics, version 24.0 (IBM Corp., Armonk, NY, USA). The distributional characteristics of the outcome variables were assessed using the Shapiro–Wilk test. The primary analysis was conducted using a two-way repeated-measures analysis of variance (ANOVA), with Time (pre-intervention and post-intervention) specified as the within-subject factor and Group [psychological skills training (PST) and control group (CG)] as the between-subject factor. The main effects of Time and Group, as well as the Group × Time interaction, were examined. Because the within-subject factor comprised only two levels, the assumption of sphericity was not applicable and therefore no sphericity correction was required. When a significant main effect or Group × Time interaction was observed, Bonferroni-adjusted pairwise comparisons were performed to identify the source of the differences. Partial eta-squared (ηp^2^) was reported as a measure of effect size, with values of approximately 0.01, 0.06, and 0.14 interpreted as small, medium, and large effects, respectively (Richardson, 2011). Degrees of freedom were reported with the corresponding F statistics. Statistical significance was set at *p* < 0.05, and 95% confidence intervals (CIs) were calculated and reported for relevant estimates.

Because chance baseline imbalances may occur in randomized trials, baseline outcome values were also examined for their potential influence on the estimated intervention effects. In addition to the primary repeated-measures ANOVA, a baseline-adjusted analysis of covariance (ANCOVA) was conducted as a robustness analysis for outcomes exhibiting a meaningful baseline imbalance between the groups. For each such outcome, the corresponding pre-intervention score was entered as a covariate, Group was specified as the between-subjects factor, and the post-intervention score was entered as the dependent variable. This analysis was used to estimate the between-group difference in post-intervention scores after statistically adjusting for baseline values and to determine whether the principal findings were robust to baseline imbalance. The adjusted analyses were considered supplementary robustness analyses and were interpreted alongside, rather than as replacements for, the primary repeated-measures ANOVA.

## 3 Result

Upon examining Table 3, the pre-test–post-test measurements of SP and DP scores were compared for the psychological skills training group and the control group (CG), and the effects of time and group × time interaction were analyzed. In terms of SP scores, it is seen that the values in the PST, which were 21.00 ± 2.83 in the pre-test, increased to 24.50 ± 2.50 in the post-test, showing an increase of approximately 16.67%. In contrast, in the CG, the scores decreased from 17.22 ± 1.63 in the pre-test to 16.33 ± 1.68 in the post-test, showing a decrease of approximately 5.16%.

**TABLE 3:** Pre–post comparison of DP and SP scores in the intervention and control groups, and results of time and group × time interaction effects.

Para-meters	Time	PST		95% CI		Δ %	CG		95% CI		Δ %	Time	Group × time	Baseline-adjusted effect
		Mean	S. D	L.B.	U.B.		Mean	S. D	L.B.	U.B.		F/ηp^2^/p	F/ηp^2^/p	F/ηp^2^/p
SP	Pre	21	2.83	19.89	22.11	16.67	17.22	1.63	16.12	18.33	−5.16	F = 7.735 ηp^2^:0.185 *p* = 0.009	F = 56.525 ηp^2^:0.624 *p* < 0.001	F = 71.352 ηp^2^: 0.684 *p* < 0.001
	Post	24.5	2.50	23.48	25.52		16.33	1.68	15.31	17.35				
DP	Pre	19.39	6.08	17.02	21.76	12.33	17.83	3.45	15.47	20.20	−7.8	F = 0.529 ηp^2^: 0.015 *p* = 0.472	F = 9.360 ηp^2^: 0.216 *p* = 0.004	F = 17.850 ηp^2^: 0.373 *p* < 0.001
	Post	21.78	5.07	19.78	23.77		16.44	2.99	14.45	18.44				

According to the statistical analysis results, the main effect of time was statistically significant for the SP variable *F*(1,34) = 7.735, *p* = 0.009, ηp^2^ = 0.185. In addition, the Group × Time interaction was statistically significant (*F* (1,34) = 56.525, *p* < 0.001, ηp^2^ = 0.624), indicating that changes in SP scores over time differed significantly between the PST and control groups. The PST group demonstrated a significant increase in self-reported perceived capacity for self-defense against simple physical attacks, whereas the control group showed a decrease.

When examining the DP scores, it is seen that the values in the PST, which were 19.39 ± 6.08 in the pre-test, increased to 21.78 ± 5.07 in the post-test, representing an increase of approximately 12.33%. In contrast, it was determined that the pre-test values in the CG, which were 17.83 ± 3.45, decreased to 16.44 ± 2.99 in the post-test, showing a decrease of approximately 7.8%. According to the statistical analysis results, the main effect of time is not significant for the DP variable (*F*(1,34) = 0.529, *p* = 0.472, ηp^2^ = 0.015). However, the group × time interaction was found to be significant (*F* (1,34) = 9.360, *p* = 0.004, ηp^2^ = 0.216) and indicates a moderate effect size. The PST group demonstrated a significant improvement in self-reported perceived capacity for self-defense against dangerous physical attacks. Overall, the findings reveal that the implemented psychological skills training program resulted in significant improvements, particularly in perceived physical self-defense capacity performance, whereas no similar improvement was observed in the control group.

The two-way repeated-measures ANOVA demonstrated significant Group × Time interactions for both SP and DP (*p* < 0.05). Importantly, the baseline-adjusted ANCOVA robustness analyses provided further support for these findings. After adjusting for baseline differences, the adjusted post-test scores remained significantly higher in the intervention group than in the control group for both SP (*F* = 71.352, ηp^2^ = 0.684, *p* < 0.001) and DP (*F* = 17.850, ηp^2^ = 0.373, *p* < 0.001). These findings indicate that the observed intervention-related differences remained robust after accounting for baseline imbalance, supporting the stability of the intervention effects across the two analytical approaches.

Table 4 shows a comparison of pre-test and post-test measurements of inhibition and initiation performance, the two sub-dimensions of self-control, for the intervention group (IG) and control group (CG). In terms of inhibition scores, it is seen that the values in the PST, which were 15.06 ± 2.55 in the pre-test, increased to 15.67 ± 2.43 in the post-test, showing an increase of approximately 4.05%. In contrast, scores in the CG decreased from 11.56 ± 1.29 in the pre-test to 9.89 ± 1.81 in the post-test, representing a 14.44% decrease. According to the statistical analysis results, the main effect of time was not significant for inhibition (*F*(1,34) = 1.456, *p* = 0.236, ηp^2^ = 0.04); however, the group × time interaction was found to be significant (*F* (1,34) = 8.172, *p* = 0.007, ηp^2^ = 0.194). This result indicates that the change observed during the intervention process differed between the two groups and that the improvement observed in the PST was more positive compared to the control group.

**TABLE 4:** Pre–post comparison of inhibition and initiation scores in the intervention and control groups, and results of time and group × time interaction effects.

Para-meters	Time	PST		95% CI		Δ %	CG		95% CI		Δ %	Time	Group × time	Baseline-adjusted effect
		Mean	S. D	L.B.	U.B.		Mean	S. D	L.B.	U.B.		F/ηp^2^/p	F/ηp^2^/p	F/ηp^2^/p
Inhibition	Pre	15.06	2.55	14.09	16.03	4.05	11.56	1.29	10.59	12.53	−14.44	F = 1.456 ηp^2^: 0.041 *p* = 0.236	F = 8.172 ηp^2^: 0.194 *p* = 0.007	F = 14.250 ηp^2^: 0.283 *p* < 0.001
	Post	15.67	2.43	14.64	16.69		9.89	1.81	8.86	10.91				
Initiation	Pre	13.11	4.03	11.63	14.49	15.25	10.33	1.68	8.86	11.81	−13.94	F = 0.268 ηp^2^: 0.008 *p* = 0.608	F = 14.157 ηp^2^: 0.294 *p* < 0.001	F = 22.680 ηp^2^: 0.415 *p* < 0.001
	Post	15.11	3.05	13.97	16.25		8.89	1.41	7.75	10.03				

When initiation performance was examined, values in the PST increased from 13.11 ± 4.03 in the pre-test to 15.11 ± 3.05 in the post-test, representing a significant increase of 15.25%. In contrast, in the CG, there was a decrease from 10.33 ± 1.68 to 8.89 ± 1.41, representing a decrease of approximately 13.94%. The main effect of time was not statistically significant for the initiation variable (*F*(1,34) = 0.268, *p* = 0.608, ηp^2^ = 0.008); however, the group × time interaction was highly significant (*F* (1,34) = 14.157, *p* < 0.001, ηp^2^ = 0.294) and indicated a medium-to-large effect size. Overall, these findings reveal that the intervention improved performance in the initiation and inhibition components of self-control functions, whereas no similar improvement was observed in the control group. This suggests that the intervention program had a positive effect on self-reported self-control processes and self-control functions.

The significant Group × Time interactions identified in the two-way repeated-measures ANOVA for inhibition (*p* = 0.007) and initiation (*p* < 0.001) were further supported by the baseline-adjusted ANCOVA robustness analyses. After statistically adjusting for baseline differences, the adjusted post-test scores remained significantly higher in the intervention group than in the control group for both inhibition (*F* = 14.250, ηp^2^ = 0.283, *p* < 0.001) and initiation (*F* = 22.680, ηp^2^ = 0.415, *p* < 0.001). These findings indicate that the observed intervention-related differences remained robust after accounting for baseline imbalance and were consistent across the primary repeated-measures ANOVA and the supplementary baseline-adjusted analyses.

Table 5 presents the pre-test–post-test results for the intervention group (IG) and control group (CG) regarding the dimensions of confidence, control, and persistence, along with the time and group × time interaction effects. When examining the confidence dimension, it is seen that the values in the PST, which were 18.17 ± 2.04 in the pre-test, increased to 21.56 ± 1.25 in the post-test, showing an increase of approximately 18.66%. In contrast, in the CG, the pre-test value of 19.89 ± 2.19 decreased to 18.89 ± 2.03 in the post-test, representing a decrease of 5.03%. According to the analysis results, the main effect of time is significant *F*(1,34) = 6.618, *p* = 0.014, ηp^2^ = 0.163; furthermore, the group × time interaction is highly significant (*F* (1,34) = 59.976, *p* < 0.001, ηp^2^ = 0.638). These findings indicate that the intervention has a strong effect on the perception of safety. When the control dimension is evaluated, it is seen that the pre-test value of 9.72 ± 1.23 in the PST increased to 12.17 ± 0.99, representing a significant increase of 25.21%. In the CG, there was a decrease from 9.83 ± 1.29 to 8.78 ± 1.44, representing a reduction of approximately 10.68%. The main effect of time was found to be significant for this variable (*F* (1,34) = 5.116, *p* = 0.030, ηp^2^ = 0.131), and the group × time interaction was also statistically significant (*F* (1,34) = 32.486, *p* < 0.001, ηp^2^ = 0.489). In the persistence dimension, values increased from 9.83 ± 0.38 to 11.94 ± 1.26 in the PST, representing a 21.46% increase. In contrast, the CG group showed a decrease from a pre-test value of 10.17 ± 0.92 to 9.89 ± 0.90, representing a decrease of approximately 2.75%. The main effect of time is significant (*F*(1,34) = 10.107, *p* = 0.003, ηp^2^ = 0.229 and the group × time interaction is also highly significant (*F* (1,34) = 32.709, *p* < 0.001, ηp^2^ = 0.490). Overall, significant improvements were observed in all three dimensions in the intervention group, while the control group showed either a decrease or limited change. These results indicate that the intervention had a significant and strong effect on participants’ perceptions of confidence, control, and persistence.

**TABLE 5:** Pre–post comparison of confidence, control, and persistence scores in the intervention and control groups, and results of time and group × time interaction effects.

Para-meters	Time	PST		95% CI		Δ %	CG		95% CI		Δ %	Time	Group × time	Baseline-adjusted effect
		Mean	S. D	L.B.	U.B.		Mean	S. D	L.B.	U.B.		F/ηp^2^/p	F/ηp^2^/p	F/ηp^2^/p
Confidence	Pre	18.17	2.04	17.15	19.18	18.66	19.89	2.19	18.88	20.90	−5.03	F = 6.618 ηp^2^: 0.163 *p* = 0.014	F = 59.976 ηp^2^: 0.638 *p* < 0.001	F = 52.798 ηp^2^: 0.615 *p* < 0.001
	Post	21.56	1.25	20.75	22.36		18.89	2.03	18.08	19.69				
Control	Pre	9.72	1.23	9.12	10.33	25.21	9.83	1.29	9.23	10.44	−10.68	F = 5.116 ηp^2^: 0.131 *p* = 0.030	F = 32.486 ηp^2^: 0.489 *p* < 0.001	F = 66.121 ηp^2^: 0.667 *p* < 0.001
	Post	12.17	0.99	11.58	12.76		8.78	1.44	8.19	9.37				
Persistence	Pre	9.83	0.38	9.49	10.17	21.46	10.17	0.92	9.83	10.51	−2.75	F = 10.107 ηp^2^: 0.229 *p* = 0.003	F = 32.709 ηp^2^: 0.490 *p* < 0.001	F = 30.527 ηp^2^: 0.481 *p* < 0.001
	Post	11.94	1.26	11.42	12.47		9.89	0.90	9.36	10.41				

All significant Group × Time interactions identified in the two-way repeated-measures ANOVA (*p* < 0.001) were further supported by the baseline-adjusted ANCOVA robustness analyses. After statistically adjusting for baseline values, the intervention-related differences remained significant across all mental toughness dimensions, with the adjusted post-test scores being significantly higher in the intervention group than in the control group for Confidence (*F* = 52.798, ηp^2^ = 0.615, *p* < 0.001), Control = 66.121, ηp^2^ = 0.667, *p* < 0.001), and Persistence (*F* = 30.527, ηp^2^ = 0.481, *p* < 0.001). These findings indicate that the observed intervention-related differences in the mental toughness dimensions remained robust after accounting for baseline imbalance and were consistent across the primary repeated-measures ANOVA and the supplementary baseline-adjusted analyses.

## 4 Discussion

The present study examined whether an 8-week structured psychological skills training program could influence self-control, perceived physical self-defense capacity, and mental resilience in professional female wrestlers. The significant Group × Time interactions observed across these domains suggest that the intervention was associated with meaningful changes in several psychological resources that are relevant to functioning in demanding combat-sport environments. Importantly, these findings should be interpreted within a broader wellbeing-oriented framework rather than exclusively as evidence of enhanced athletic performance. Contemporary perspectives on combat sports emphasize that their psychological significance extends beyond competitive outcomes to include resilience, self-regulation, confidence, empowerment, social and psychological health, and safe participation, while also recognizing that potential benefits depend on the conditions in which combat sports are practiced (Bandura, 1997; Harwood-Gross et al., 2021; Ng-Knight et al., 2021). The present findings therefore provide preliminary experimental evidence that a structured psychological intervention embedded within wrestling training may contribute to selected psychological resources relevant to athlete development and perceived wellbeing.

This interpretation is particularly relevant for female wrestlers. Self-control, perceived self-defense self-efficacy, confidence, perceived control, and persistence may represent complementary psychological resources through which athletes manage demanding training and competitive situations. Improvements in these domains may support perceived agency and adaptive coping, although the present study did not directly measure global psychological wellbeing, quality of life, or long-term participation. Accordingly, the findings should not be interpreted as demonstrating a direct improvement in overall wellbeing. Rather, they indicate that PST may strengthen specific psychological resources that are theoretically relevant to wellbeing and sustainable participation in combat-sport settings.

The improvements observed in simple and dangerous perceived physical self-defense capacity may also be relevant from an empowerment perspective. However, because the Physical Self-Defense Scale assesses perceived capacity rather than objectively demonstrated self-defense proficiency, the findings should be interpreted as changes in perceived competence and preparedness rather than evidence of improved physical self-defense skill. Within a wellbeing-oriented combat-sport framework, enhanced perceived self-defense self-efficacy may nevertheless represent a potentially meaningful psychological resource because confidence in one’s ability to respond to challenging situations may contribute to perceived agency and autonomy. This interpretation is consistent with the broader conceptualization of combat sports as practices that may promote psychological resources and empowerment when delivered within structured, safe, and ethically appropriate environments (Ciaccioni et al., 2025a,b, 2026). Future studies should determine whether changes in perceived self-defense self-efficacy are accompanied by objective behavioral measures of self-protection, decision-making, or sport-specific responses under controlled conditions.

The improvements observed in SP and DP scores should be interpreted as changes in participants’ perceived physical self-defense capacity rather than objectively demonstrated self-defense proficiency. The Physical Self-Defense Scale captures participants’ perceptions of their capacity to respond to simple and dangerous physical attacks; it does not provide a behavioral or performance-based assessment of actual self-defense skills. Therefore, the present findings suggest that PST may positively influence athletes’ perceived confidence or preparedness in relation to physical self-defense situations, but they do not establish that the intervention improved objectively measured self-defense ability. Future studies should incorporate validated behavioral or observational self-defense assessments to determine whether changes in perceived capacity are accompanied by measurable improvements in physical self-defense performance.

Although the randomized controlled design and significant Group × Time interactions provide evidence that changes over time differed between the PST and control groups, the findings should not be interpreted as demonstrating that the observed improvements were exclusively caused by the specific active components of PST. The control condition consisted of routine wrestling training and was not attention-matched to the supervised psychological intervention. Consequently, nonspecific effects related to additional intervention contact, attention, expectancy, and social interaction cannot be completely excluded. Moreover, the relatively small sample size and baseline imbalances reduce the precision with which intervention effects can be estimated. Although baseline-adjusted supplementary analyses yielded results consistent with the primary analyses, these limitations warrant cautious interpretation of the magnitude and specificity of the observed effects.

The present results are generally consistent with the available literature, although direct evidence specifically examining physical self-defense outcomes remains limited. The strongest support comes from the randomized controlled trial conducted by Yıkılmaz et al. (2025), in which a structured intervention similarly improved both simple and dangerous perceived physical self-defense capacity in women compared with a control group. Although this represents one of the few studies directly evaluating self-defense outcomes, it provides important evidence that structured psychological or integrated mental training can positively influence behavioral responses relevant to self-protection and combat performance.

Indirect evidence from performance-based interventions also supports the present findings. A meta-analysis examining pressure training across sport, military, and law-enforcement settings reported a moderate overall improvement in operational performance (*g* = 0.67), indicating that psychologically demanding training environments can enhance performance under stress (Low et al., 2020). Likewise, Jones et al. (2022) demonstrated that a brief mental skills intervention increased observer-rated mental toughness and improved military training performance after controlling for physical fitness and leadership environment. Collectively, these findings suggest that psychological skills training may improve performance in high-pressure situations by facilitating attentional regulation, emotional control, and adaptive behavioral responses.

Nevertheless, the current evidence should be interpreted with appropriate caution. A recent meta-analysis reported that although psychological skills training generally produces moderate benefits compared with control conditions, these effects become less consistent when analyses are restricted to studies with stronger methodological quality (Reinebo et al., 2023). Similarly, a systematic review found that while approximately 90% of published reviews concluded that PST was beneficial, nearly all were judged to be of critically low methodological quality (Lange-Smith et al., 2023). Furthermore, findings from defense and law-enforcement populations remain heterogeneous. For example, Voigt and Zinner (2023) reported that adding simulated stress to knife-defense training did not produce superior outcomes compared with standard training, suggesting that the effectiveness of psychologically demanding interventions may depend on the nature of the task and training context.

Despite these limitations, the present study extends the existing literature by providing randomized controlled evidence that an 8-week psychological skills training program was associated with improvements in participants’ perceived capacity for self-defense against simple and dangerous physical attacks in professional female wrestlers. Given the scarcity of experimental research investigating self-defense-related psychological outcomes in combat sports, these findings provide novel evidence supporting the integration of structured psychological skills training into athlete development programmes. Future multicenter randomized trials incorporating objective behavioral and competition-performance measures are warranted to confirm the generalizability and long-term sustainability of these effects.

The present findings demonstrate that the 8-week psychological skills training program significantly improved both inhibition and initiation components of self-control in professional female wrestlers compared with routine training alone. The significant group × time interaction effects indicate that the PST group showed greater improvements in self-reported inhibition and initiation components of self-control than the control group. These findings suggest that the intervention may have influenced participants’ perceived ability to regulate behavioral responses and initiate goal-directed actions, as reflected in their self-reported self-control scores. However, these findings should not be interpreted as evidence of objectively improved self-reported inhibition component of self-control, self-reported self-control, or decision-making performance.

The relevance of these findings may extend beyond competitive performance. Self-regulation is a central psychological resource for maintaining adaptive behavior under demanding conditions, and its development may be particularly valuable in combat sports, where athletes must repeatedly regulate emotional responses, attention, impulses, and behavior within a physically and psychologically demanding environment. Nevertheless, the present findings reflect changes in self-reported self-control and should not be interpreted as evidence of objectively improved executive functioning. From a wellbeing perspective, the findings suggest that structured psychological training may provide athletes with additional self-regulatory resources that could support adaptive coping within the training environment, while the extent to which these changes translate into broader psychological health or long-term wellbeing requires direct investigation.

The present results are largely consistent with the growing body of literature indicating that structured psychological interventions and combat sport training can improve self-regulation, inhibitory control, and related psychological outcomes (Moore et al., 2023; Wang and Liu, 2025; Özkan et al., 2026; Zheng et al., 2026). Among the available evidence, the randomized controlled trial by Zheng et al. (2026) provides the closest support to the present findings, reporting significant group × time effects for self-regulation following a structured martial arts intervention. Importantly, that study complemented self-report measures with objective assessments of self-reported inhibitory self-control using the Stroop task, suggesting that improvements in psychological self-regulation may also translate into measurable enhancements in executive control.

Evidence regarding specific self-control subcomponents, however, remains less consistent. Köroğlu et al. (2025) demonstrated that judo training significantly improved behavioral initiation but did not produce significant changes in inhibition, whereas other intervention studies have reported gains in one or both executive control domains depending on the characteristics of the training programme (Harwood-Gross et al., 2021; Ng-Knight et al., 2021). Compared with these studies, the present findings showing concurrent improvements in both inhibition and initiation suggest that the multidimensional content of the psychological skills training programme–including goal setting, self-talk, imagery, attentional control, emotional regulation, and pre-performance routines–may have produced broader adaptations in self-regulatory functioning than interventions focusing primarily on sport-specific practice alone.

Several mechanisms may explain these improvements. Wrestling is characterized by rapidly changing competitive situations requiring continuous attentional regulation, emotional control, behavioral inhibition, and rapid initiation of appropriate motor responses (Özkan et al., 2026; Ponomarev et al., 2025). Psychological skills training directly targets many of these executive processes through repeated practice of cognitive and behavioral strategies. Moreover, interventions that progressively challenge executive functioning through repeated self-regulatory demands have consistently been associated with improvements in executive control and behavioral regulation (Diamond and Lee, 2011; Harwood-Gross et al., 2021). Recent evidence syntheses also suggest that combat sport participation produces relatively consistent improvements in inhibitory control, even when broader mental health outcomes remain more heterogeneous (Ciaccioni et al., 2023).

Nevertheless, the current evidence should be interpreted cautiously. Although the superiority of psychological skills training over routine training is increasingly supported, relatively few randomized controlled trials have simultaneously examined both inhibition and initiation within combat sport populations. Consequently, direct comparisons across studies remain difficult because intervention content, outcome measures, and participant characteristics differ considerably. Future multicenter randomized trials incorporating objective neurocognitive assessments alongside psychometric measures would provide a more comprehensive understanding of the mechanisms through which psychological skills training enhances self-control. Overall, the present findings extend the current combat sport literature by demonstrating that an 8-week psychological skills training programme can simultaneously improve both inhibition and initiation components of self-control in professional female wrestlers. Given the central role of self-regulation in tactical self-regulatory functioning, emotional stability, and competitive performance, these findings support the integration of structured psychological skills training into long-term athlete development programmes as a complement to physical and technical preparation.

In the study, significant increases were observed in the intervention group from pre-test to post-test in the mental resilience sub-dimensions of confidence, control, and persistence among female wrestlers, while decreases or limited changes emerged in the control group. The analysis results indicated that the main effect of time was significant for all three variables and that the group × time interaction was significant for confidence (*F* = 59.976, *p* < 0.001, ηp^2^ = 0.638), control (*F* = 32.486, *p* < 0.001, ηp^2^ = 0.489), and persistence (*F* = 32.709, *p* < 0.001, ηp^2^ = 0.490) dimensions. These findings reveal that the intervention program implemented produced a meaningful and strong improvement in participants’ perceptions of confidence, control, and persistence.

These findings are generally consistent with the existing literature indicating that structured psychological interventions can enhance key components of mental resilience, including confidence, perceived control, and persistence in athletes. Previous studies have reported that relatively short-term psychological training programs often produce meaningful improvements in self-confidence, emotional regulation, and resilience-related outcomes, whereas control groups typically demonstrate minimal changes over comparable periods. In particular, psychological interventions implemented in competitive sport settings appear to facilitate athletes’ ability to cope with performance-related stressors, maintain focus under pressure, and sustain adaptive psychological functioning during training and competition (Bordo et al., 2025; Reinebo et al., 2023).

Within combat sport contexts, psychological skills-based interventions have shown promising effects on psychological outcomes closely related to performance, including self-confidence, emotional stability, anxiety regulation, and coping effectiveness. However, the available evidence remains relatively limited, particularly when compared with other sporting disciplines, and high-quality experimental studies involving female combat sport athletes are still scarce (Harwood-Gross et al., 2021). Therefore, the present findings contribute novel evidence supporting the role of psychological skills training in promoting resilience-related psychological characteristics among professional female wrestlers.

The magnitude and direction of the observed changes are also supported by previous intervention studies. For example, Oguntuase and Sun (2022) reported that an 8-week mindfulness-based intervention significantly improved resilience and self-confidence in elite soccer players compared with a non-intervention control group. Similarly, Bordo et al. (2025) demonstrated that an 8-week practice-based psychological intervention improved self-confidence and arousal regulation among competitive tennis players, whereas no meaningful changes were observed in controls. Comparable findings have also been reported in adolescent athletes, where an 8-week mindfulness intervention resulted in significant improvements in overall resilience and perceived control, with benefits persisting for several months after the intervention period. Furthermore, psychological skills and mindfulness-based training programs implemented in futsal players have been associated with enhanced mental toughness, improved coping strategies, and greater self-confidence following the intervention period. Collectively, these findings suggest that structured psychological interventions lasting approximately 8–10 weeks may be sufficient to induce meaningful improvements in resilience-related psychological capacities across different athletic populations.

Nevertheless, the current findings should be interpreted in light of several important considerations. Although the broader literature generally supports the effectiveness of psychological interventions in sport, the overall strength of evidence remains heterogeneous. A large-scale meta-analysis by Reinebo et al. (2023) reported that the beneficial effects of sport psychology interventions were attenuated when analyses were restricted to studies with stronger methodological designs. Likewise, Lange-Smith et al. (2023) concluded that although the vast majority of reviews reported positive effects of psychological skills training on performance-related outcomes, most of the available evidence was judged to be of critically low methodological quality. These observations highlight the continued need for rigorously designed randomized controlled trials with adequate sample sizes, transparent reporting procedures, and long-term follow-up assessments.

Despite these limitations, the present results align with one of the most consistently reported findings in the sport psychology literature: targeted psychological skills training appears to enhance resilience-related characteristics beyond the effects of routine athletic training alone. The significant Group × Time interactions indicate that changes in self-reported inhibition and initiation differed between the PST and control groups. These findings are consistent with a beneficial effect of the PST intervention on self-regulatory tendencies as reflected by the self-control questionnaire. However, the present findings should not be interpreted as evidence of objectively improved executive inhibition, cognitive control, or competitive decision-making performance, because these domains were not directly assessed using behavioral or neurocognitive measures. The observed changes therefore represent improvements in participants’ self-reported self-control rather than objectively demonstrated changes in executive functioning. Future studies should combine psychometric measures with validated behavioral inhibition tasks, neurocognitive assessments, and sport-specific decision-making measures to determine whether changes in perceived self-regulation translate into objectively measurable behavioral adaptations.

### 4.1 Limitations

The findings of this study reveal important results regarding the effects of a structured psychological skills training program implemented with professional female wrestlers on self-control, self-defense self-efficacy, and mental resilience; however, certain methodological limitations should be considered. Firstly, the sample size of the study is relatively limited and consists solely of female wrestlers aged 18–25. This may limit the generalizability of the findings to different age groups, male athletes, or other combat sports. Future studies conducted with larger and more heterogeneous samples will contribute to a more comprehensive assessment of the intervention’s effects on different sports disciplines and athlete profiles. Secondly, the variables assessed in the study were predominantly determined by self-report measurements based on psychometric scales. Although the validity and reliability properties of the scales used are supported in the literature, self-report-based measurements may be influenced by participants’ perceptual biases or social desirability effects. Therefore, in future research, the use of neurophysiological and biological indicators (e.g., heart rate variability, EEG measurements, or stress hormones) in addition to the assessment of psychological variables may contribute to a more objective understanding of the mechanisms of intervention effects. Another limitation is that the intervention program was limited to 8 weeks, and no long-term follow-up measurements were conducted in the study. Moreover, the control group received routine wrestling training rather than an attention-matched psychological intervention. Therefore, nonspecific effects associated with additional attention, expectancy, social interaction, or contact with the intervention provider cannot be completely excluded. Therefore, it was not possible to assess whether the effects of psychological skills training applications on athletes’ psychological skills are sustainable in the long term. Future studies incorporating longer intervention periods and follow-up measurements will be important in determining the sustainability of the gains achieved. However, although the intervention protocol was implemented within a structured program framework in this study, it should not be overlooked that individual differences, such as participants’ personal music preferences, personal motivation levels, or psychological characteristics, may potentially influence the intervention effect. Finally, although the results obtained in the study revealed significant effects on psychological performance variables, direct sports performance indicators (e.g., competition performance, technical success rate, or physical performance tests) were not evaluated within the scope of the research. Therefore, it could not be definitively established how the psychological skills training intervention directly reflected in competitive performance alongside psychological development. Future studies that include performance and physiological parameters in addition to psychological measurements will contribute to a more comprehensive understanding of the holistic effects of psychological skills training applications on sports performance.

An important measurement-related limitation is that all primary outcomes were assessed using self-report instruments. Consequently, the observed improvements in perceived physical self-defense capacity and self-control should not be interpreted as evidence of objectively demonstrated changes in self-defense proficiency, self-reported inhibition component of self-control, self-reported self-control, or decision-making performance. The Physical Self-Defense Scale assesses participants’ perceived capacity to respond to physical attacks, whereas the self-control measure assesses self-reported inhibition and initiation tendencies. Neither instrument provides direct behavioral or performance-based assessment. Therefore, future research should combine self-report measures with objective, improved self-reported inhibition tasks, neurocognitive assessments, validated sport-specific performance tests, and, where appropriate, observational assessments of self-defense behavior. Such multimethod designs would help determine whether improvements in perceived psychological capacities translate into objectively measurable behavioral or performance changes.

A further limitation concerns the possibility of attention, expectancy, and social-interaction effects. Participants in the PST group received weekly supervised sessions, daily psychological activities, and greater structured interaction with the intervention team, whereas the control group continued routine wrestling training without an attention-matched intervention. Although participants were informed that the program was intended to support training preparation rather than specifically improve the study outcomes, expectancy effects cannot be completely excluded. Moreover, all outcomes were assessed using self-administered self-report measures, making the findings potentially susceptible to response and reporting biases. Therefore, the observed between-group differences may reflect not only the specific effects of the PST components but also nonspecific effects associated with increased attention, engagement, and intervention contact. Future studies should address this limitation by incorporating attention-matched active control conditions and combining self-report measures with objective behavioral, neurocognitive, physiological, and sport-specific performance outcomes. Overall, despite these limitations, the study presents important experimental findings on the effects of psychological skills training-based psychological interventions on self-control, self-defense, and mental resilience in female wrestlers and contributes to the growing body of psychological skills training-based psychological intervention approaches in the sports psychology literature.

### 4.2 Future research

The findings of this study indicate that psychological skills training interventions have positive effects on self-control, self-defense, and mental resilience in female wrestlers; however, certain limitations and gaps in the literature present significant opportunities for future research. Firstly, comparative studies involving different age groups, genders, and combat sports would enhance the generalizability of psychological skills training’s effects. Furthermore, long-term interventions and follow-up measurements could analyze the persistence and sustainability of effects on athletes’ psychological skills. Future research could also examine the effects of different modalities and intensities of psychological skills training on psychological and performance outcomes. Furthermore, explaining the mechanisms of psychological effects through biological and neurophysiological indicators (e.g., heart rate variability, EEG, stress hormone levels) will contribute to a better understanding of how psychological skills training works in the sports context. Finally, obtaining robust evidence through multicenter and randomized controlled trials, both experimentally and practically, will support the feasibility of using psychological skills training as a standard intervention method in holistic psychological development strategies for athletes.

### 4.3 Practical implications

The practical implications of this study should be considered within a context-sensitive combat-sport and wellbeing framework. First, coaches and sport psychologists may consider integrating structured psychological skills training into wrestling programs as a complementary component of athlete development, particularly when the objective is to support self-regulatory resources, perceived competence, confidence, and adaptive coping. Such interventions should not be viewed as substitutes for technical, physical, or tactical preparation, but rather as complementary strategies embedded within the broader training process. Second, the potential psychological benefits of PST are likely to depend on how the intervention is implemented. Consistent with recent conceptual work on combat sports and wellbeing, implementation should occur within structured and ethically appropriate environments that prioritize athlete safety, respectful coach–athlete relationships, individualized support, and appropriate monitoring of psychological demands (Ciaccioni et al., 2025a,^2026^). For female wrestlers in particular, interventions addressing perceived self-defense self-efficacy and confidence may also contribute to perceived agency and empowerment, although these outcomes should be evaluated directly in future research rather than inferred from psychological performance measures alone. Third, practitioners should avoid assuming that participation in combat sports or psychological training inherently produces wellbeing benefits. The quality of coaching, training climate, safety procedures, intervention fidelity, athlete characteristics, and opportunities for autonomy may all influence outcomes. Therefore, sustainable implementation should involve appropriately trained practitioners, clear intervention protocols, regular monitoring, and adaptation to athletes’ individual needs. These considerations are consistent with the emerging emphasis on prevention, protection, structured environments, instructor practices, and sustainable development within contemporary combat-sport research (Ciaccioni et al., 2026).

## 5 Conclusion

The findings indicate that an 8-week psychological skills training program was associated with significant improvements in self-control, perceived physical self-defense capacity, and mental toughness among professional female wrestlers. Improvements were also observed across the relevant dimensions of these psychological constructs, including confidence, persistence, and control. These findings suggest that psychological skills training may be a useful complementary approach for developing psychological skills and perceived self-defense capacity in female wrestlers. However, because the present study did not directly assess competitive performance, technical success, or objectively measured physical self-defense ability, the observed psychological improvements should not be interpreted as evidence of enhanced wrestling performance or objectively demonstrated self-defense proficiency. Furthermore, the small sample, passive control condition, self-report measures, baseline imbalances, and absence of follow-up warrant cautious interpretation of the magnitude and durability of the observed effects. Future studies should incorporate competition-based performance indicators, sport-specific technical assessments, and objective physical self-defense measures to determine whether improvements in psychological characteristics translate into measurable performance outcomes.

## Data Availability

The raw data supporting the conclusions of this article will be made available by the authors, without undue reservation.
